# Pathways to Aging: The Mitochondrion at the Intersection of Biological and Psychosocial Sciences

**DOI:** 10.4061/2011/814096

**Published:** 2011-09-26

**Authors:** Martin Picard

**Affiliations:** Department of Kinesiology and Physical Education, McGill University, 475 Pine Avenue, Montreal, QC, Canada H2W 1S4

## Abstract

Compelling evidence suggests that both biological and psychosocial factors impact the process of aging. However, our understanding of the dynamic interplay among biological and psychosocial factors across the life course is still fragmentary. For example, it needs to be established how the interaction of individual factors (e.g., genetic and epigenetic endowment and personality), behavioral factors (e.g., physical activity, diet, and stress management), and psychosocial experiences (e.g., social support, well-being, socioeconomic status, and marriage) in perinatal, childhood, and adulthood influence health across the aging continuum. This paper aims to outline potential intersection points serving as an interface between biological and psychosocial factors, with an emphasis on the mitochondrion. Mitochondria are cellular organelles which play a critical role in cellular senescence. Both chronic exposure to psychosocial stress and genetic-based mitochondrial dysfunction have strikingly similar biological consequences; both predispose individuals to adverse age-related health disorders and early mortality. Exploring the interactive nature of the factors resulting in pathways to normal healthy aging, as well as those leading to morbidity and early mortality, will continue to enhance our ability to translate research into effective practices that can be implemented throughout the life course to optimise the aging process.

## 1. Introduction

 Aging is the inescapable process by which individuals, from the age of about 30 years old onwards, gradually lose maximal functional capacity [1]. Some resilient individuals experience a slow decline lasting several decades, attaining ages past one hundred years old and more [2]. These are exceptional centenarians who experience minimal physical impairment [3] along with healthy minds and bodies [4, 5]. However, many individuals experience more rapid functional declines in their 60's or 70's, sometimes afflicted with the “frailty syndrome”—defined as a lack in general strength and unusual susceptibility to disease or to other infirmity [6]—and these individuals often suffer from multiple age-related morbidities such as cardiovascular disease, neurodegenerative diseases, diabetes, and cancer [7]. The majority of individuals lie between these two extreme scenarios, with an average life expectancy of 81 years old in North America [8]. 

In the past century, we have witnessed significant increases in life expectancy as more individuals live longer [9, 10]. This increase in average life expectancy has undoubtedly resulted from advances in medical technologies and preventive medicine that prevent most (>80%) early deaths due to acute illnesses (e.g., infections and injuries) and prolong life of individuals afflicted with chronic life-threatening conditions (e.g., HIV/AIDS and cardiovascular disease) [10]. The outcome of effectively delaying mortality is that morbidity is postponed—or “compressed”—to older ages, as described in Fries' *compression of morbidity hypothesis* [11]. The incidence of age-related diseases has remained stable over the last decades, or even increased, which significantly contributes to health care costs [10]. Given that an unprecedentedly large proportion of the population is expected to reach 60–80 years of age in the next two decades [12], changes in political and social health policy will be necessary to face societal challenges [10, 13]. Comprehensive frameworks including the panoply of factors capable of potently modulating the human aging process may be essential to address the impending social imperative of implementing health-enhancing strategies for the elderly, at minimal costs.

A central question concerning longevity remains: Why do some people live long whereas others die early? Another equally critical question concerns morbidity: Why is aging associated with a greater incidence of almost every categorized disease—including degenerative, metabolic, and malignant disorders? Since disease incidence, mortality, and longevity are all associated terms in the same aging equation, a more general question may be posed: What are the pathways that impact individuals' rate of aging? 

While it is well understood that both biological and psychosocial factors impact the aging process, it is still unclear *how* psychosocial factors influence cellular aging and translate into aging of the whole organism [4]. Below is a selective review focusing on physiological systems susceptible to constitute critical convergence points, acting as integrators of the interactive forces imposed by both biological and psychosocial factors. Understanding how this physiological integration takes place will improve researchers' means to develop multilevel interventions that optimize the decline in physical function associated with aging.

Physical health and function depends on the coordinated functioning of several organs and physiological systems that allow the organism in dynamic balance to adapt to perpetual environmental challenges. Failure to adapt to challenges (e.g., healing wounds, increasing energy expenditure, and replenishing dying postmitotic cells) may occur in aging. Thus, senescence-induced loss of cell numbers and/or optimal functioning can result in suboptimal organ function [14]. For this reason, markers of cellular aging, such as nuclear DNA telomere length, is occasionally used as an indicator of aging. These are the protective caps at the end of chromosomes, whose reduction in length is often used as a reliable and proximal indicator of cellular senescence [15].

## 2. Biological Determinants of Aging—A Role for the Mitochondrion?

Biological factors influence the aging process. An important constituent of mammalian cells are mitochondria. These dynamic subcellular organelles contain their own circular DNA, are the principal site of cellular adenosine triphosphate (cellular energy currency) synthesis, regulate cell death through apoptotic signalling, and are the major source of reactive oxygen species (ROS) within the cell [16, 17]. One of the most scrutinized hypothesis in aging research is the mitochondrial theory of aging which stipulates that, over time, mitochondrial DNA accumulates oxidative damage from ROS, which negatively impacts mitochondrial function, leading to cellular dysfunction, organ failure, and ultimately results in age-related disease [14]. Data supporting this theory has been obtained from transgenic animals with enhanced protection against mitochondrial oxidative damage [18, 19]. These mice, which over-express a mitochondrial-targeted catalase, are resistant to age-related insulin resistance [18] and have slightly increased lifespan [19]. However, existing data render this theory imperfect [20–23] and evidence supporting a direct role of ROS in aging has largely been correlative [24]. Furthermore, examples exist in vertebrates (e.g., naked mole rat [25]) and invertebrates (*c. elegans* [26, 27]) where the typical negative correlation between ROS production and lifespan is uncoupled. Although ROS-induced damage has not consistently been causally linked to aging, a general shift in intra- and extracellular redox state towards more oxidized levels occurs in aging cells and in the blood of aged individuals, which could have important implications for redox-sensitive signalling pathways and their influence on the aging process [28, 29].

In contrast, a general role of mitochondria in the aging process is supported by abounding experimental evidence [30–37]. For example, animals with a deficient proof-reading version of the mitochondrial DNA polymerase gamma (PolG mutator mice), a defect which leads to an abnormally rapid accumulation of mitochondrial DNA mutations, exhibit several characteristics reminiscent of an accelerated aging phenotype (e.g., graying of fur, loss of muscle and brain mass, and kyphosis) [38, 39]. This indicates that mitochondrial DNA damage is capable of causing aging-like symptoms such as organ dysfunction and early mortality. It must be noted that whether this model actually mimics natural human aging is uncertain [40, 41]. Similarly, whether ROS dictates the aging process [23] remains a contentious issue. Nevertheless, although its exact cellular and physiological impact remain unclear, the integrity of mitochondrial DNA is challenged during aging [42, 43] and may contribute to cellular senescence and consequently, to the progressive functional changes in organs that characterize the aging process. 

Further evidence supporting a role of mitochondria in the aging process comes from interventions that influence mitochondrial function. The only intervention capable of extending life span in animals—lifelong caloric restriction [44]—diminishes damage to mitochondrial DNA and concomitantly decreases the age-related decline in muscle aerobic capacity [45, 46]. Of note, caloric restriction has also been reported to decrease the incidence of age-related illnesses (e.g., cancer) in rodents [47], providing an interesting empirical link between mitochondrial integrity and age-related morbidity. Moreover, evolutionarily inherited single nucleotide polymorphisms yielding genetic variants of mitochondrial DNA, called haplogroups, may influence mitochondrial function and health outcomes in humans (reviewed in [14]). Indeed, mitochondrial haplogroups have been associated with mitochondrial ROS production and cellular oxidative capacity [48–50], resting metabolic rate and energy expenditure in humans [51], and disease incidence, progression, and longevity [14, 52–54]. Collectively, this suggests that intrinsic mitochondrial factors (e.g., related to mitochondrial DNA) can indeed influence the aging process. 

Like caloric restriction, physical activity and exercise are potent stimuli that increase mitochondrial content and function [55–57]. Physical activity reduces the age-related decline in function of different organ systems including brain and muscles. Indeed, it is established that individuals who are more physically active exhibit lower incidences of age-related diseases and mortality [58–60] as well as better control of existing chronic diseases [60]. Furthermore, endurance exercise prevents the premature aging-like characteristics of the PolG mutator mice including mitochondrial abnormalities, skeletal muscle, and brain atrophy [61]. The converse is also true. Physical *inactivity* leads to a reduction in mitochondrial content and function [56, 62] and contributes towards insulin resistance (i.e., prediabetic state) [63] and enhanced metabolic risk [64]. 

The aforementioned physiological dysregulations are more commonly observed in old age. For example, older individuals (63–70 years old) who are sedentary, but not those who are active, have lower mitochondrial content than young individuals [65]. Finally, dietary lipid supply (e.g., virgin olive oil) has been shown to impact membrane composition in brain mitochondria and to reduce oxidative damage to these organelles with aging in rats [66]. Thus, factors that impact mitochondrial function (i.e., levels of physical activity, caloric restriction, and diet) can consequently impact age-related disease incidence, progression, and survival. 

The findings outlined in this section are consistent with the notion that biological mechanisms determine the aging process. Additional arguments supporting this notion also exist. They notably include the loss of molecular fidelity with time as the major cause of aging [67] and the recently discovered link between mitochondrial function, telomere length, and cellular senescence [68, 69]. Because each aspect outlined above appears to modulate the aging process in small yet sizeable ways, we must acknowledge that evidence suggests that the rate of aging is not solely determined by single biological factors, such as how many calories are ingested, which genetic polymorphism an individual has inherited, and how much physical activity is performed. Rather, in real-life situations, the rate of aging for a given individual must ultimately be determined by the dynamic and reciprocal interplay of these and many other factors, as discussed below.

## 3. Psychosocial Determinants of Aging

Despite the fact that aging research has generally been dissected using the biological scalpel, psychological and social variables are also important modulators of the aging process associated with mortality [70–73]. For example, personality and lifestyle may influence longevity in humans [4]. In a prospective study of patients with coronary heart disease, the authors found that pessimism and anxious personality traits were associated with adverse age-related health outcomes such as greater cancer incidence [74] and all-cause mortality [75]. Degradation in negative affect (i.e., more negative emotions) was also a strong prognostic indicator of long-term mortality in coronary heart disease patients [76], suggesting that negative emotions can adversely influence survival and resilience. 

On the other hand, centenarians with engaged lifestyle and certain personality traits (e.g., emotional stability, extraversion, and openness) tend to have higher mental health status, a healthy sign of aging, when compared to those who do not possess these traits [77]. A twenty-year prospective population study showed that individuals with more positive self-perceptions of aging tended to live about seven years longer than those with less positive perceptions of aging [78]. Likewise, self-rated health—SRH, how an individual subjectively rates his/her health—is one of the most powerful statistical predictor of morbidity and mortality [79, 80]. Of note, SRH is often a more powerful statistical predictor of mortality than clinical and biological assessments of health. Similarly, high socioeconomic status is associated with more positive multisystemic physiological profiles (i.e., allostatic load), which predict lower morbidity and mortality rates with aging [81, 82]. There is also evidence that “protective” psychosocial factors such as control beliefs and quality of social support (i.e., emotional links with family and friends) contribute to better maintenance of functional capacity with aging [83]. Although not directly supporting a causal link between psychosocial factors and longevity, these data strongly suggest that several psychosocial factors impact physiological pathways to aging and distal outcomes such as mortality and longevity.

Psychosocial factors also have similar effects on more proximal biological indices of aging. Not living with a partner (i.e., being unmarried) is associated with accelerated cellular aging, as evidenced by shorter telomere length in blood leukocytes of unmarried middle-aged men and women [84]. Similar reports by Epel and colleagues demonstrate that psychological stress is associated with accelerated telomere shortening [85]. Likewise, depression has been linked with accelerated rates of aging and cellular senescence [86] and mortality [75], demonstrating that psychosocial forces may accelerate cellular aging [72]. Collectively, these findings indicate that psychosocial forces can exert both negative and positive influences on the aging process, affecting both morbidity and mortality.

## 4. Mitochondria: Interfacing Two Worlds

As mentioned above, mitochondria influence cellular function [16, 17] and impairments in mitochondrial function due to genetic variations/mutations [14] or other stresses such as physical inactivity [56] may accelerate the aging process. Interestingly, several hormones including those involved in the body' stress responses to psychosocial stressors modulate the synthesis of new mitochondria (mitochondrial biogenesis) and can modify important parameters of mitochondrial function [87]. Indeed, mitochondrial DNA transcription and mitochondrial biogenesis are modulated by the glucocorticoid hormone cortisol downstream from the hypothalamic-pituitary-adrenal (HPA) axis, by catecholamines secreted by the sympathetic-innervated adrenal medulla (epinephrine and norepinephrine), thyroid hormones, and by the steroid hormone estrogen, as well as by several cytokines (e.g., IL-1*α*, IL-1*β*, and TNF*α*) [88]. 

**Figure 1:**
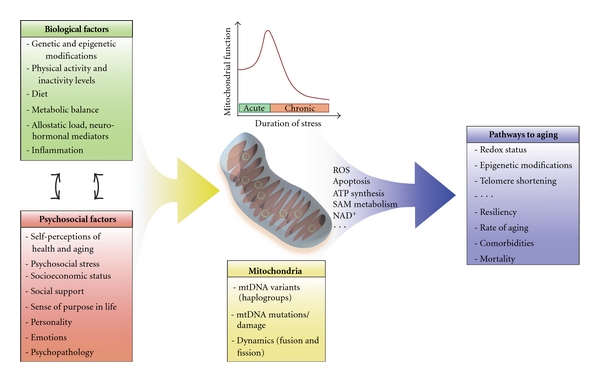
Mitochondria influence pathways to aging by operating at the intersection of biological and psychosocial factors. Biological and psychosocial factors dynamically/bidirectionally interact to influence mitochondrial content and function in the body's tissues. The same factors can exert opposite effects on mitochondrial function, depending on the duration of exposure. For instance, acute stressors tend to upregulate mitochondrial biogenesis and function, whereas chronic stressors tend to downregulate mitochondrial biogenesis and function (top center graph). Mitochondrial-level factors influence mitochondrial function and may determine mitochondrial responsivity to upstream biological and psychosocial influences. In response to multiple individual and environmental factors, mitochondria produce outputs influencing cellular function, gene expression, and cellular senescence. As a result, pathways to aging are ultimately determined by the integrated and synergistic influence of multiple biological and psychosocial factors.

In fact, the mitochondrial DNA sequence contains putative response elements for several hormonal receptors (e.g., thyroid and glucocorticoid hormones, and insulin) [89] and some receptors for glucocorticoids, thyroid hormones, and estrogen have even been found in mitochondria of different cell types [90, 91]. Acutely, these “stress” hormones increase mitochondrial biogenesis and function [87]. However, chronic exposure to elevated levels of these hormones, which can be induced by psychosocial stressors (e.g., social isolation, depression, and violent or abusive social environment) [93], can lead to reductions in mitochondrial mass (see Figure 1) and concomitant increases in mitochondria-derived ROS production [87, 92]. These mitochondrial outputs synergistically damage cellular components and contribute to cellular senescence when chronically produced at high levels. 

Beyond the direct effects that psychosocial stresses exert on mitochondrial function, psychosocial factors can also influence individual's lifestyles, such as levels of physical activity and inactivity (i.e., sedentariness) [93]. For example, negative perceptions of one's body image and negative influence from family and friends are associated with lower levels of physical activity [95–97]. Similarly, people who are depressed or suffer from mental illnesses also tend to be more physically inactive [98, 99]. Physical inactivity can in turn undermine physical and mental health [100], predisposing inactive young individuals to depression later in life [101]. 

Physical activity has positive effects on mitochondrial function and counteracts inflammatory processes and age-related chronic diseases [55]. It can even buffer against the negative effects of chronic stress on telomere shortening [102]. In fact, the beneficial effects of physical activity and exercise on the hormonal system (e.g., increases in interleukin 6, growth hormone, brain-derived growth factor—BDNF) [103, 104], on psychological and cognitive aspects (e.g., decrease in stress levels and reactivity to stress, depression, improved well-being) [105], as well as on metabolic regulation (e.g., increased mitochondrial mass and improved insulin sensitivity) [65, 106], suggest that exercise and physical activity exert multisystemic protective effects which can prevent the deleterious consequences of chronic stress [107]. Indeed, improving physical fitness has been shown to decrease hormonal, physiological and psychological markers of chronic stress [72]. Psychosocial factors and physical activity can therefore interact to influence mitochondrial function and modulate the impact of chronic stress on the body.

Because mitochondria influence cellular aging and are responsive to stress hormone levels, they are especially well equipped to act as key integrators that synergistically influence biological and psychosocial factors (Figure 1). As described above, work in the psychosocial sciences has unravelled important links between how individuals feel, their social contexts, and the effects of these factors on mortality and longevity. However, how these factors influence and interact with biological factors remains to be explored in more depth. The findings described above and many others bring new evidence coaxing researchers to focus on the *interactions* of biological and psychosocial forces that influence the aging process [70]. Conclusions derived from research *not* adopting an integrative approach risk being uni-dimensional and thus difficult to apply towards different real-life contexts, where individuals age under the collective influence of factors of different nature.

## 5. Interdisciplinarity: A Necessary Framework for Aging Research?

Interdisciplinarity and even transdisciplinarity [108] have emerged as key necessities in the field of aging and others [108–110]. Both the National Institutes of Health (NIH) in the USA and the Canadian Institutes of Health Research (CIHR) funding agencies have opened institutes on/of aging promoting broad mandates that necessarily reach across traditional disciplinary boundaries. Although some problems are best addressed with the approach of a single discipline, other issues require the integration of several disciplines to fully comprehend the complexity of the processes at play [111]. This is particularly true for aging [109]. Likewise, the discipline of developmental psychopathology, driven by the impetus to grasp and impact the complexities of mental health at different stages of development, has evolved to become a deeply “interdisciplinary field that seeks to elucidate the interplay among the biological, psychological, and social-contextual aspects of normal and abnormal development across the life course” [112, p.16]. The writings of Cicchetti and Toth [112] are particularly enlightening regarding the conceptual, theoretical, and practical directions to take to achieve such a degree of integration within a discipline. They particularly stress the importance of cross-disciplinary dialogue [112], along with the need for an emphasis on the process of development naturally resulting from the interdependence over time of multiple biological and psychosocial factors [113].

In this spirit, the aging process is influenced by several interactive forces inherent to the individual (e.g., genetic endowment, physical activity, diet, lifestyle, and personality) and forces inherent to the psychosocial environment (e.g., sociocultural context, family, and life stress) that continuously and progressively interact over long periods. Aging must therefore be approached from an ontogenic perspective not solely focused on end outcomes (i.e., mortality, comparison of aged and young individuals) or on the molecular factors predicting these outcomes. Instead, aging research would benefit from being guided by a perspective focused on the changes and interactions among biological and psychosocial processes, which take place across stages of human development throughout the lifespan.  Table 1 lists five conceptual propositions elaborated by Ryff and Singer [70] to advance hypothesis-testing research focused on healthy aging as an interdisciplinary process. In a similar way that mental health and disease emerge from cumulative life experiences in infancy, childhood, and adulthood [112–114], aging must also be a deeply experience-dependent process where the biology influences how a person responds to their experiences, but where the biology is also shaped by those experiences.

## 6. Experience-Dependent Modulation of Aging: A Role for Epigenetics?

The experience-dependent nature of aging has important implications for the research questions that are posed (e.g., How do specific events/factors at different life stages interact to modulate the rate of aging?). This suggests that cumulative prenatal, earlylife, young adulthood, and later life circumstances impact pathways to aging. This could be mediated in part by alterations in the stress system across the life course: induced alterations in neurological substrates that signal stressful information [114, 115] as well as neurobiological and allostatic processes involving inflammation and oxidative stress [72, 116]. Thus, as suggested by Epel [72], psychological and metabolic stress may constitute a potent recipe for accelerated cellular aging. 

Epigenetics, which involves the laying of relatively stable imprints on the genome that impact gene expression and cellular function over time [117, 118], is increasingly revealed as a candidate intersection point between biological and psychosocial processes in several age-related chronic diseases [119, 120]. It is known that epigenetic marks are altered in aging [121, 122] and in several age-related disease states such as cancer, neurodegenerative, and autoimmune diseases [123], as well as type 2 diabetes [124, 125]. The altered epigenome could therefore mediate the experience-dependent modulation of the aging rate and age-related morbidities across the life span. Further to that point, mitochondria themselves possess a plastic mtDNA epigenome [126] and have the potential to generate powerful signals capable of affecting the nuclear epigenome [127, 128], making these organelles well equipped to play a critical interfacing role between the environment and the genome [127]. Although this remains to be empirically supported, it is an hypothesis that integrates knowledge about the health consequences attributable to genetic variations, calorie intake, physical activity/inactivity, and neurobiological substrates of psychosocial stress into a unified framework for aging research. 

Taken together, the reviewed literature indicates that an increasing number of biological factors (e.g., mtDNA haplotypes, hormones, genetic polymorphisms affecting cellular signalling pathways, factors epigenetic imprints), behavioral and (e.g., diet/calorie intake, and exercise), and psychosocial (e.g., psychological stress, depression, personality, and marital status) factors influence the aging process. The challenge lying ahead of researchers in this field lies in the exploration of the intersection points linking these multiple levels of analysis spanning several disciplines. For example, what physiological processes interact with the psychosocial effects of being married, of meditating regularly, or of experiencing psychological well-being and sense of purpose in life, which ultimately culminate in reduced telomere shortening [129]? What combination of elements lead to resilience and successful adaptation to aging? And what are the combinations leading to age-related risk and ill-health? Interdisciplinary initiatives aimed at describing the interactive biopsychosocial processes that link these multiple levels will yield new knowledge of the pathways to aging, which in turn will inform effective prevention and intervention strategies. Network perspectives inspired from systems biology [130, 131] allowing modeling of complex nonlinear interactions among the studied variables may prove useful in this endeavour. Similarly, building comprehensive theories of aging will require the combined efforts of researchers from different disciplines contributing diverse complementary expertise, perspectives, and approaches to study aging.

## 7. Individualized Aging Trajectories

Effective strategies for promoting healthy aging will need to be individualized. The perfect individualization of treatment and prevention of age-related disorders may appear as an unattainable utopia at this point in time. This is particularly the case because up until now, our knowledge of the dynamic interplay between the different biological and psychosocial levels of analysis is still fragmentary, which impedes discoveries about the complex processes from which individual-specific pathways of aging emerge. A well-known principle in biology and developmental psychopathology is that of “equifinality,” whereby multiple distinct pathways lead to the same outcome. The reciprocal principle is that of “multifinality,” whereby the same set of pathways lead to different outcomes [112]. Likewise, the source of interindividual differences in aging trajectories undoubtedly lies in the interplay of several interdependent pathways of which there is no single universal “right” combination that can be prescribed.

Means must be developed to distinguish between optimal (i.e., living to your full biological potential) and suboptimal (i.e., dying or having disease sooner than your constitution should permit) rates/trajectories of aging. From the onset, it can be established that optimal aging is characterized by a slow progressive decline in physiological functions, maintenance of well-being for the majority of the lifespan, and only a short period of very poor physical health leading to death. But what are the biomolecular (i.e., gene expression, mitochondrial function, and biomarkers) sings of optimal adaptation to the passing decades? What are the normative ontogenic trajectories, or healthy biological and physiological signatures of successful aging? Having answers to these questions will enable researchers to more accurately distinguish dysfunction from normal function in different aged organ systems. Ryff, Singer and colleagues have established biological correlates and a conceptual framework aimed at deciphering the biological and psychosocial underpinnings of resilience, positive health, and successful aging [71, 132–134]. Building such a knowledge base of normal molecular, cellular, physiological, and psychosocial signatures of aging may also translate into more refined means to detect predisease or preclinical deviations from normal adaptation and to prevent age-related diseases. 

Thus far, despite the fact that more resources are being invested to study specific aspects of the normal pathways leading to healthy aging [71, 132–134], relatively little data is available to address pressing questions about healthy aging. A noteworthy exception is the MacArthur Studies of Successful Aging, which have collected a rich dataset spanning multiple biological and psychosocial levels over several years, thus providing an exceptional design for longitudinal evaluation of the biological-psychosocial interactions for a large cohort of elderly individuals [135]. Future smaller-scale (i.e., intervention trials and animal-based models) integrative research initiatives should build from the strengths and experience of this and other such longitudinal endeavours [136].

## 8. Conclusions

In conclusion, as a rejoinder to the question “What are the pathways that impact individuals rate of aging?”, we ought to answer that there are surely several different pathways to healthy aging. These pathways must depend not on singular factors acting independently, but on interactive forces among multiple levels of function operating in synergy [108], including biological, behavioural, psychosocial and spiritual factors [108]. Identifying the developmental nature of “pathways to aging” is an interdisciplinary task inviting researchers in aging to join forces to discover and refine our comprehension of the intersections between our respective disciplines. Biomedical scientists need to appreciate the complexity of biological-psychosocial interactions involved in health processes; and psychosocial researchers need to appreciate the underlying biological factors susceptible to modulate individual responses to psychosocial challenges. This can be achieved, along with the ensuing collaborative interdisciplinary successes in research, by defining and empirically testing potential intersection points among biological and psychosocial disciplines. A deeper understanding of these intersections, and of the ensuing mind-body cross-talk [92], will enhance our appreciation of the multiple interacting facets that collectively determine optimal and suboptimal rates of aging for individuals. Testing and defining inter- and transdisciplinary intersection points should also enhance our ability to translate health discoveries into applicable interventions to promote the health and quality of life of an increasingly old population.

## Figures and Tables

**Table 1:** Research propositions to advance knowledge of healthy aging—Adapted from Ryff and Singer [70].

Proposition 1	*Health promotion processes:* positive psychosocial factors predict better biological regulation
	*Premise:* positive health and high levels of well-being are associated with lower morbidity, decreased physical symptoms and pain, increased longevity, increased resistance to illness, decreased stroke incidence, and better glycemic control.

Proposition 2	*Resilience Processes:* positive psychosocial factors protect against the damaging effects of external adversity
	*Premise:* psychological strengths (e.g., personality traits and coping ability) and favourable social situations (e.g., social/family support and high socioeconomic status) are associated with “physiological toughness” and an enhanced ability to maintain a high-level of functioning in the face of adversity.

Proposition 3	*Recovery and repair processes:* Positive psychosocial factors facilitate the regaining of functional and/or biological capacities
	*Premise:* hopeful individuals with optimistic beliefs and positive expectations about their health have better prognosis from heart surgeries, some cancers and HIV/AIDS, and possibly better DNA repair mechanisms.

Proposition 4	*Compensation processes:* psychological or biological strengths can offset the negative health consequences of psychological or biological weaknesses
	*Premise:* psychological distress and adversity can be moderated by positive psychological traits (e.g., coping strategies and affective styles).

Proposition 5	*Gene expression processes:* psychosocial factors as mitigating against the negative and promoting the positive
	*Premise:* many people with genetic susceptibilities to certain diseases never develop them; psychosocial and other environmentallydriven epigenetic factors may modulate genetic susceptibility to disease and gene expression patterns that impact health in aging.
